# Physiological implications of biased signaling at histamine H2 receptors

**DOI:** 10.3389/fphar.2015.00045

**Published:** 2015-03-10

**Authors:** Natalia Alonso, Carlos D. Zappia, Maia Cabrera, Carlos A. Davio, Carina Shayo, Federico Monczor, Natalia C. Fernández

**Affiliations:** ^1^Laboratorio de Patología y Farmacología Molecular, Instituto de Biología y Medicina ExperimentalBuenos Aires, Argentina; ^2^Consejo Nacional de Investigaciones Científicas y TécnicasBuenos Aires, Argentina,; ^3^Laboratorio de Farmacología de Receptores, Cátedra de Química Medicinal, Facultad de Farmacia y Bioquímica, Universidad de Buenos AiresBuenos Aires, Argentina; ^4^Instituto de Investigaciones Farmacológicas – Universidad de Buenos Aires – Consejo Nacional de Investigaciones Científicas y TécnicasBuenos Aires, Argentina

**Keywords:** 7TMR, H_2_R ligands, biased agonism, pluridimensional efficacy, GPCR internalization, ERK, HDC

## Abstract

Histamine mediates numerous functions acting through its four receptor subtypes all belonging to the large family of seven transmembrane G-protein coupled receptors. In particular, histamine H2 receptor (H2R) is mainly involved in gastric acid production, becoming a classic pharmacological target to treat Zollinger–Ellison disease and gastric and duodenal ulcers. H2 ligands rank among the most widely prescribed and over the counter-sold drugs in the world. Recent evidence indicate that some H2R ligands display biased agonism, selecting and triggering some, but not all, of the signaling pathways associated to the H2R. The aim of the present work is to study whether famotidine, clinically widespread used ligand acting at H2R, exerts biased signaling. Our findings indicate that while famotidine acts as inverse agonist diminishing cAMP basal levels, it mimics the effects of histamine and the agonist amthamine concerning receptor desensitization and internalization. Moreover, the treatment of HEK293T transfected cells with any of the three ligands lead to a concentration dependent pERK increment. Similarly in AGS gastric epithelial cells, famotidine treatment led to both, the reduction in cAMP levels as well as the increment in ERK phosphorylation, suggesting that this behavior could have pharmacological relevant implications. Based on that, histidine decarboxylase expression was studied by quantitative PCR in AGS cells and its levels were increased by famotidine as well as by histamine and amthamine. In all cases, the positive regulation was impeded by the MEK inhibitor PD98059, indicating that biased signaling toward ERK1/2 pathway is the responsible of such enzyme regulation. These results support that ligand bias is not only a pharmacological curiosity but has physiological and pharmacological implications on cell metabolism.

## Introduction

Histamine is a biogenic amine synthesized from L-histidine by histidinede carboxylase (HDC). It plays an important role in human health and disease by acting through four receptor subtypes (H1R–H4R) that differ in their expression, molecular characteristics, signal transduction pathways, and function (Hill et al., 1997; Leurs et al., 2009). Nevertheless, all types of histamine receptors belong to the seven transmembrane spanning family of G-protein coupled receptors (GPCRs). The observation that histamine-evoked gastric acid secretion could not be blocked with classical antihistamines, led to the conclusion that H2 histamine receptors (H2R) were involved in gastric acid secretion (Black et al., 1972).

Until now, one of the most clinically relevant uses of histamine receptor ligands are achieved through the regulation of H2R, which are widely expressed in most tissues (Bakker et al., 2002). Acid-related diseases affect the quality of life of patients and are important causes of morbidity and mortality (Shin et al., 2008). Approximately 40% of adults in the USA complain of monthly, 20% of weekly, and approximately 7% of daily heartburn (Sandler et al., 2002), making gastroesophageal reflux disease (GERD) one the most common gastrointestinal (GI) disorders with great monetary costs for patients and public health system (Cappell, 2005). H2 antagonists have proved to be very active agents for the treatment of Zollinger–Ellison syndrome, duodenal and gastric ulcers, reflux and esophagitis. They have an excellent safety profile that supports their common use as over-the-counter medications. Over the past decades, there have been important advances in the treatment of acid-related disorders. The discovery of proton pumps for controlling gastric acid secretion and the successful synthesis of proton pump inhibitors in the 1980s, made these drugs to emerge as the treatment of choice for acid-related diseases. However, current guidelines recommend prescribing gastroprotective agents to patients taking non-steroidal anti-inflammatory drugs (NSAIDs) to prevent GI ulcers causing a rebirth of the clinical use of H2R antagonists, specifically famotidine. A fixed-dose combination of the NSAID, ibuprofen and the H2R antagonist, famotidine (ibuprofen/famotidine; DUEXIS^®^) is available for the symptomatic treatment of arthritic symptoms and to reduce the risk of GI ulcers in patients requiring ibuprofen therapy. The use of the combination of these two drugs was shown to reduce the risk of ulcers by 50% compared with ibuprofen alone (Laine et al., 2012).

In most tissues, including the parietal cells of the gut, H2R stimulation increases adenylate cyclase activity and induces cAMP accumulation (Soll and Wollin, 1979). However, increasing evidence indicates that receptors exist as conformational collections where each conformation promotes different downstream effects. In this context, a ligand is able to cause differential activation of some signaling events associated to a particular receptor causing receptor bias (Ghanouni et al., 2001). Biased agonism has been mainly studied regarding adrenergic receptors. It has been described that carvedilol, which is used for certain cardiovascular diseases, blocks the deleterious effects mediated by β1AR and also presents and additional cardioprotective effect mediated by MAPK activation through a β-arrestin and EGFR transactivation pathway (Kim et al., 2008). Recent evidence indicates that this phenomenon of biased signaling may be extended to H2R inverse agonists cimetidine, ranitidine, and tiotidine (Reher et al., 2012; Alonso et al., 2014).

Given the novel indication for famotidine, in the present study we try to establish whether this drug, previously classified as inverse agonist for its negative efficacy on adenylate cyclase activity, displays positive efficacy regarding receptor desensitization, internalization, or adenylate-cyclase-independent signaling. Our results showed that famotidine induces H2R desensitization in transfected HEK293T cells, leading to receptor down-regulation. Furthermore, all H2R ligands tested, famotidine, histamine, and amthamine (H2R agonist) induced ERK1/2 activation not only in HEK293T cells, but also in an endogenous expression system, human gastric adenocarcinoma cells. Remarkably, ERK phosphorylation promoted by all ligands assayed induces HDC expression in AGS cells.

In this work we demonstrate that the H2R inverse agonist famotidine displays positive efficacy regarding receptor desensitization, internalization, and ERK activation. Finally, our findings may have relevant clinical implications given that famotidine regulates HDC expression and that this ligand is used clinically in long term treatments.

## MATERIALS AND METHODS

### Materials

Cell culture medium, antibiotics, isobutylmethyl xanthine (IBMX), cAMP, bovine serum albumin (BSA), cycloheximide, amthamine, famotidine, forskolin, and PD98059 were obtained from Sigma Chemical Company (St. Louis, MO, USA). Tiotidine were from Tocris Cookson Inc. (Ballwin, MO, USA). [^3^H]cAMP, and [^3^H]tiotidine were purchased from Perkin Elmer Life Sciences (Boston, MA, USA). Fetal calf serum was from Natocor (Argentina). Other chemicals used were of analytical grade and obtained from standard sources.

### Plasmid Constructions

GRK2, -3,-5, and -6 cDNAs were subcloned into the pCEFL vector (pCEFLGRK2,-3, -5, and -6) and the human H2R was subcloned previously in in our laboratory (Shayo et al., 2001) in the pCEFLHA vector (pCEFLHA-H2R).

### Cell Culture

HEK293T (Human embryonic kidney) and AGS (human gastric cancer) cells were cultured in Dulbecco’s modified Eagle’s medium (DMEM) and Kaighn’s Modification of Ham’s F-12 medium (F12K), respectively, supplemented with 10% fetal calf serum and 5 μg/ml gentamicin at 37^∘^C in humidified atmosphere containing 5% CO_2_.

### Transient Transfection

For transient transfection of HEK293T, cells were grown to 80–90% confluency. cDNA constructs were transfected into cells using K2 Transfection System (Biontex, Munich, Germany). The transfection protocol was optimized as recommended by the supplier. Assays were performed 48 h after transfection and the expression of the constructs was confirmed by immunobloting using specific antibodies.

### cAMP Assays

For concentration-response assays, cells were incubated 3 min in basal culture medium supplemented with 1 mM IBMX at 37^∘^C, followed by 9 min exposure to different concentrations of the ligands. For desensitization assays, cells were pretreated with 10 μM H2R ligands in the absence of IBMX for different periods of time as shown in the figures. Cells were thoroughly washed and resuspended in fresh medium containing 1 mM IBMX, incubated for 3 min, and exposed to 10 μM amthamine for 9 min to determine whether the system was able to generate a cAMP response. In all experiments, the reaction was stopped by ethanol addition followed by centrifugation at 2000 ×*g* for 5 min. The ethanol phase was then dried and the residue resuspended in 50 mM Tris-HCl pH 7.4, 0.1% BSA. cAMP content was determined by competition of [^3^H]cAMP for PKA, as previously described (Davio et al., 1995).

### Radioligand Binding Assay

Saturation binding experiments were carried out by incubating the cells for 40 min with increasing concentrations of [^3^H]tiotidine, ranging from 0.4 up to 240 nM in the absence or presence of 1 μM unlabeled tiotidine. The incubation was stopped by dilution with 3 ml of ice-cold 50 mM Tris-HCl pH 7.4 and the bound fraction was collected in 200 μl of ethanol. Experiments on intact cells were carried out at 4^∘^C to avoid ligand internalization. The kinetic studies performed with 2 nM [^3^H]tiotidine at 4^∘^C showed that the equilibrium was reached at 30 min and persisted for 4 h (data not shown).

### Receptor Internalization and Recovery

HEK293T cells were incubated at different times with 10 μM famotidine and the number of receptor sites was analyzed by radioligand binding assay. The recovery of binding sites was evaluated by saturation binding assays at 60 min after thoroughly washing the cells previously exposed to 10 μM famotidine for 90 min. In assays performed with 50 μM cycloheximide, the inhibitor was added 30 min before ligand treatment.

### Western Blot Assays

For Western blot assays, cells were lysed in 50 mM Tris-HCl pH 6.8, 2% SDS, 100 mM 2-mercaptoethanol, 10% glycerol, and 0.05% bromophenol blue and sonicated to shear DNA. Total cell lysates were resolved by SDS-PAGE, blotted and incubated with the primary antibodies anti-, -ERK1/2, -pERK, -GRK2, 3, 5, and 6 (Santa Cruz Biotechnology, CA, USA), followed by horseradish peroxidase conjugated anti-rabbit or anti-mouse (Santa Cruz Biotechnology, CA, USA) and developed by enhanced chemiluminescence (ECL) following the manufacturer’s instructions (Amersham Life Science, England). Films were scanned and quantified using Scion Image^®^ software from National Institutes of Health (NIH).

### RT-PCR and Quantitative Real-time PCR

Total RNA was isolated from AGS cells using Quick-Zol reagent (Kalium Technologies) following the manufacturer’s instructions. For the first-strand cDNA synthesis, 1 μg of total RNA was reverse-transcribed using the High Capacity cDNA Reverse Transcription kit (AB) with random primers. Quantitative real-time PCR (qPCR) was performed using 1 μL of the resulting cDNA, amplified at 45 cycles for 15 s at 94^∘^C, 20 s at melting temperature (60^∘^C), and 30 s at 72^∘^C using the HOT FIREPol EvaGreen qPCR Mix Plus (Solis Biodyne). Quantitative PCR was performed in triplicate using the Rotor Gene Q detection system (Qiagen) and the following primers: human HDC forward, 5′-GGACAAAGACAACTGGTGTGCC-3′ and reverse, 5′-AATGGTTAGCACGGTGCAGTGG-3′; and human β-Actin (βAct) forward, 5′-GGACTTCGAGCAAGAGATGG-3′ and reverse 5′-AGCACTGTGTTGGCGTACAG-3′ as described in Melgarejo et al. (2006). The specificity of each primer set was monitored by analyzing the dissociation curve, and the relative HDC mRNA quantification was performed using the comparative ΔCt method using Actin as the housekeeping gene.

### Statistical Analysis

Statistical analysis was performed from at least three independent experiments. Binding data, sigmoidal dose-response, desensitization fittings, and comparison of best fit values according to extra-sum of squares *F* test were performed with GraphPad Prism 5.00 for Windows, GraphPad Software (San Diego, CA, USA). One-way ANOVA followed by the Dunnett’s post-test was performed using GraphPadInStat version 3.01, GraphPad Software (San Diego, CA, USA). Specific binding was calculated by subtraction of non-specific binding from total binding. Statistical of densitometric western blot analysis were carried out by one-way ANOVA or *t*-test followed by the Dunnett’s or Tukey’s Multiple Comparison post-test performed with GraphPad Prism 5.00 for Windows, GraphPad Software.

## Results

### Famotidine Induced H2R Desensitization and Internalization

**FIGURE 1:**
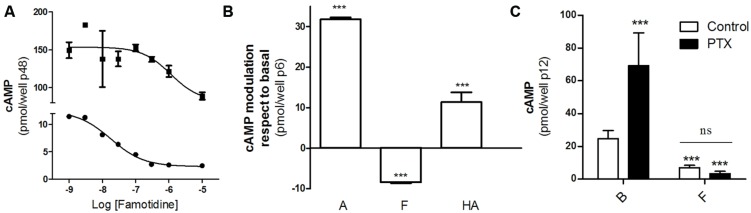
Negative efficacy of famotidine. **(A)** H2R transfected HEK293T cells pretreated (■) or not (∙) with 25 μM forskolin were exposed for 9 min to increasing concentrations of famotidine at 37^∘^C in the presence of 1 mM IBMX. **(B)** Cells were exposed for 9 min to 10 μM amthamine (A), 10 μM famotidine (F) or 100 μM histamine (HA) at 37^∘^C in the presence of 1 mM IBMX. **(C)** Cells were pretreated for 6 h with (*black bars*) or without (*open bars*) 100 ng/ml pertussis toxin (PTX) and exposed to 10 μM famotidine (F) for 9 min, in the presence of 1 mM IBMX. ^∗∗∗^*p* < 0.001 with respect to basal (B); ns*,* no significant difference. **(A–C)** Cyclic AMP levels were determined as detailed under Experimental Procedures. Data were calculated as the means ± SD of assay duplicates. Similar results were obtained in at least three independent experiments. Error bars are not visible when their size is smaller than the symbol.

We first studied the effect of famotidine on cAMP accumulation in HEK293T cells transfected with the H2R. According to literature, famotidine behaves as an H2R inverse agonist so it was expected that diminishes cAMP levels. **Figure 1** shows that famotidine reduced basal cAMP levels in a concentration-dependent fashion in both forskolin pretreated or untreated cells (**Figure 1A**), and that both amthamine and histamine were able to stimulate adenylyl cyclase (**Figure 1B**), confirming the already described efficacy of these H2 ligands in our transfection system. To exclude the possibility that the H2R couples to a G-protein inhibitory of adenylyl cyclase (Gαi) we performed similar assays in presence of pertussis toxin (PTX). Since famotidine ability to diminish cAMP levels was not affected by PTX pretreatment we conclude that it behaves as an inverse agonist dampening H2R constitutive activity, establishing the negative efficacy of the ligand regarding cAMP regulation in our model of study (**Figure 1C**).

**FIGURE 2:**
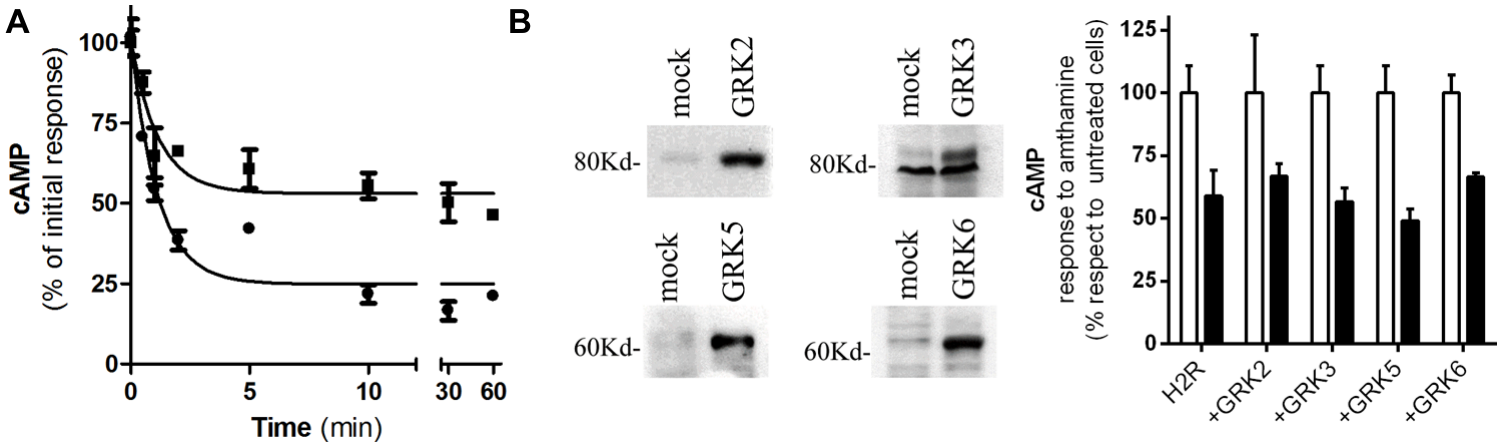
Famotidine induced H2R desensitization. **(A)** H2R-transfected HEK293T cells were exposed to 10 μM famotidine (∙) or 10 μM amthamine (■) for different time periods, then washed, and cAMP response to amthamine was determined. **(B)** Cells were transfected with H2R or co-transfected with different GRKs. (Left) Western blot analysis of the expression of the different GRKs. (Right) Cells were pretreated for 10 min with 10 μM famotidine (*black bars)* or not* (open bars),* washed and exposed for 9 min to 10 μM amthamine in the presence of 1 mM IBMX. **(A,B)** Cyclic AMP levels were determined as detailed under Experimental Procedures and expressed as the difference between the stimulus to the agonist and basal cAMP levels respect to the response of control cells without treatment. Data were calculated as the means ± SD of assay triplicates. Similar results were obtained in at least four independent experiments. Error bars are not visible when their size is smaller than the symbol.

We have previously reported that amthamine and H2R inverse agonists, ranitidine, and tiotidine, were able to promote receptor desensitization and internalization; however, cimetidine showed to promote receptor internalization but not significant receptor desensitization. Based on that, we aimed to evaluate whether famotidine induces H2R desensitization and internalization. With this purpose, H2R transfected HEK293T cells were exposed to 10 μM famotidine at different time periods. After carefully washing, cells were re-challenged with amthamine and cAMP response was evaluated. The cAMP response evoked by amthamine in cells previously exposed to famotidine for 1 h was 53 ±7% of the initial response achieved by cells without pre-treatment. Although the extent of receptor desensitization was lower than the evoked by amthamine (Fernandez et al., 2008), famotidine induced a significant H2R desensitization (**Figure 2A)**. Co-transfection of HEK293T cells with the H2R and each of the most ubiquitous members of GRK family of proteins (GRK2, 3, 5, and 6) did not alter the degree of H2R desensitization indicating that GRK family is not involved in the desensitization evoked by famotidine (**Figure 2B)**. It is worth noting that GRK2 is involved in amthamine induced H2R desensitization in U937 cells, and COS7 and HEK293T heterologous transfection systems (Fernández et al., 2002; Fernandez et al., 2011) while GRK3 proved to desensitize H2R response to amthamine in COS7 co-transfected cells (Shayo et al., 2001).

**FIGURE 3:**
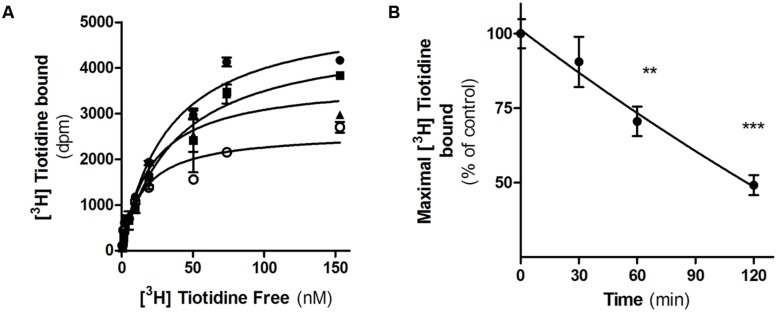
Famotidine induced H_2_R internalization. (A) H_2_R-transfected HEK293T cells were exposed or not (∙) to 10 μM famotidine for 30 min (■), 60 min (▲), or 120 min (**∘**) and H2R binding sites were determined by saturation assays as described under Experimental Procedures. **(B)** Data represent the percentage maximal bound value fitted by non-linear regression of [^3^H]Tiotidine saturation assay. Data were calculated as the means ± SD of assay duplicates. Similar results were obtained in at least three independent experiments. ^∗∗^*p* < 0.01; ^∗∗∗^*p* < 0.001 with respect to untreated cells. Error bars are not visible when their size is smaller than the symbol.

We next evaluated H2R internalization by measuring the number of cell surface [^3^H]-tiotidine binding sites after incubating cells with famotidine and carefully washing. Saturation binding assays showed that famotidine treatment of HEK293T transfected cells led to a significant time-dependent reduction in the number of H2R binding sites (**Figure 3**). Following 60 min of famotidine exposure showed H2R membrane site internalization by 55% ±7%.

**FIGURE 4:**
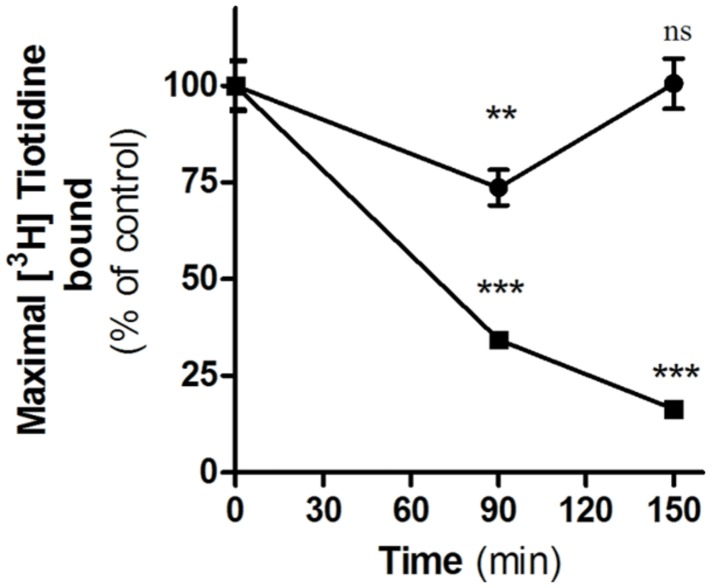
Internalization and recovery of H2R membrane sites. [^3^H]Tiotidine saturation assays were performed in H2R-transfected HEK293T cells treated for 90 min with 10 μM famotidine, washed, and further incubated for 60 min in fresh medium. Data represent the percentage of maximal bound value fitted by non-linear regression of [^3^H]Tiotidine saturation assay, calculated as the means ±SE (*n* = 3). Assays were carried out in the absence (∙) or presence of cycloheximide 50 μM (■). 100% correspond to untreated cells. ^∗∗^*p* < 0.01; ^∗∗∗^*p* < 0.001; ns*,* no significant difference with respect to untreated cells. Error bars are not visible when their size is smaller than the symbol.

We have previously described that unlike amthamine treatment, H2R internalized after inverse agonist treatment does not recycle to the plasma membrane (Alonso et al., 2014). To determine whether this observation might be extended to famotidine, we evaluated the recovery of H2R membrane sites in the presence or absence of the protein synthesis inhibitor, cycloheximide. To do this, HEK293T transfected cells exposed to famotidine for 90 min were washed and incubated during 60 min in fresh medium. Although the removal of the ligand led to a rapid recovery of the number of H2R binding sites, pretreatment with cycloheximide, completely abolished the recuperation of surface receptors (**Figure 4**). These findings indicate that, as previously described for other H2R inverse agonists, the presence of H2R sites in the plasma membrane following the removal of famotidine was a consequence of *de novo* H2R protein synthesis and not due to H2R recycling (Alonso et al., 2014).

### Famotidine Modulates ERK1/2 Phosphorylation

**FIGURE 5:**
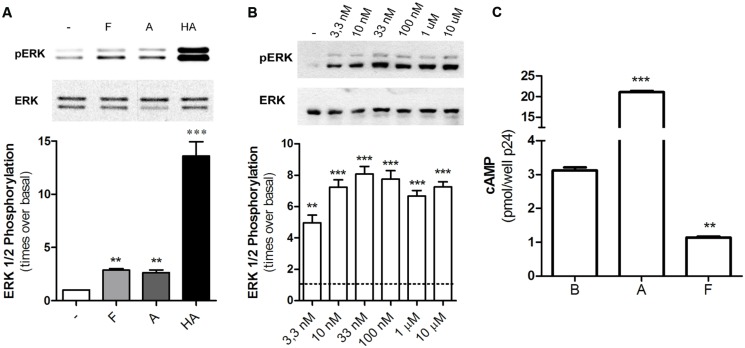
Famotidine promoted ERK phosphorylation. (**A)** H2R-transfected HEK293T cells were treated with 10 μM famotidine (F), 10 μM amthamine (A), or 100 μM Histamine (HA) for 5 min, lysed, and equal amounts of proteins were subjected to SDS-PAGE and analyzed by Western Blot. **(B)** Cells were exposed for 5 min to increasing concentrations of famotidine and western blot analysis were performed as mentioned above. (Right) Densitometric analysis of ERK phosphorylation at 5 min of treatment, normalized to the corresponding ERK total levels, obtained with the Scion Image Program. Data are expressed as times over basal p-ERK levels. Data are expressed as means ± SE (*n* = 3). ^∗∗∗^*p* < 0.001; ^∗∗^*p* < 0.01 respect to basal levels. **(C)** cAMP was determined in untreated cells **(B)** or following exposure to 10 μM amthamine (A) or 10 μM famotidine (F) for 5 min.

Diverse GPCRs activate MAPK pathways through G-protein dependent or independent mechanisms; in particular, H2R ligands have proved to increase ERK phosphorylation through a dynamin or Gβγ pathway (Shenoy and Lefkowitz, 2005; Werry et al., 2005). When we evaluated MAPK modulation in HEK293T transfected cells we found that famotidine led to a rapid increase in p-ERK levels being the maximum activation observed at 5 min following ligand treatment (**Figure 5A**). Concentration response assays showed that even the exposure to 3.3 nM of famotidine induced a significant increase in p-ERK levels even though no significantly modulation of cAMP was observed (**Figure 5B**). Taking into account that, at the times assayed, amthamine and histamine are able to increase cAMP levels while famotidine diminishes it (**Figure 5C**), it can be stated that regardless the efficacy displayed toward the Gs-AC-cAMP pathway, famotidine presents positive efficacy respect to the MEK/ERK cascade, thus behaving as an agonist toward this signaling pathway. The discrepancy observed between concentrations required to modulate cAMP and p-ERK levels, and the opposed efficacy observed by famotidine for modulation of both pathways reinforce the biased behavior of the ligand.

### Famotidine Bias in Human Gastric Adenocarcinoma AGS Cells

**FIGURE 6:**
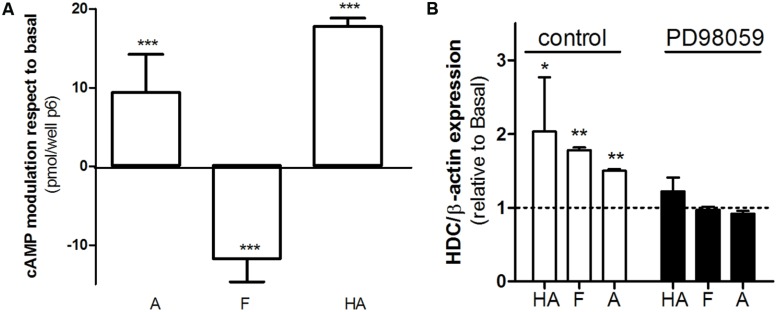
Biased signaling of famotidine. **(A)** AGS cells were exposed to 10 μM amthamine (A), 10 μM famotidine (F), or 100 μM histamine (HA) for 9 min, in the presence of 1 mM IBMX. Cyclic AMP levels were determined as detailed under Experimental Procedures. Data were calculated as the means ± SD of assay duplicates. Similar results were obtained in at least three independent experiments. **(B)** Histidine decarboxylase (HDC) gene expression was determined by quantitative real time PCR in AGS cells treated for 24 h with 10 μM amthamine (A), 10 μM famotidine, or 100 μM histamine (HA) in the presence *(black bars)* or absence of MEK inhibitor PD98059 *(white bars)*. Relative HDC mRNA quantification was performed using β-actin as housekeeping gene. ^∗^*p* < 0.05; ^∗∗^*p* < 0.01; ^∗∗∗^*p* < 0.001; respect to basal.

AGS cells represent a relevant model concerning H2R histaminergic ligands and their clinical use, and have been extensively used to evaluate histamine action and gastric acid secretion regulation (Guo et al., 2013; Ku et al., 2014). Regarding cAMP accumulation histamine and amthamine displayed positive efficacy while famotidine significantly reduced cAMP basal levels (**Figure 6A**). Once again, all three ligands assayed displayed positive efficacy toward ERK1/2 modulation. Since it has been described that ERK activity is involved in the modulation of HDC gene expression (Wessler et al., 2000; Colucci et al., 2001), we evaluated whether the agonism of famotidine toward ERK pathway modulates HDC gene expression. We found that, as well as described for histamine, amthamine and more remarkably famotidine also increased HDC expression, (**Figure 6B**). Our findings clearly show that famotidine, classically classified as antagonist or inverse agonists based on cAMP modulation may induce ERK1/2 phosphorylation not only in overexpression models, but also in human gastric adenocarcinoma cells that endogenously express H2R, with potential functional consequences since they are able to modulate the expression of the enzyme responsible for histamine synthesis.

## Discussion

Over the past few years, a growing number of articles have been published describing the identification of biased agonists at a wide variety of GPCRs, including several worldwide marketed drugs (Kenakin and Miller, 2010; Bock et al., 2014). However, although much has been speculated regarding the potential advantages of this pharmacological feature, and there are several biased agonists entering clinical studies, until now there are no drugs prescribed because of its biased behavior. Nevertheless, in spite of the intended use, ligands acting as biased agonists/antagonists are being administered everyday, and the observed adverse/unwanted effects should be also interpreted in the light of this knowledge.

We and others reported the biased signaling of H2R blockers in heterologous transfected cells as well as in naïve cell systems (Reher et al., 2012; Alonso et al., 2014). Likewise the other ligands, in H2R transfected HEK293T cells famotidine acts as an inverse agonist regarding cAMP intracellular levels, but as an agonist with positive efficacy when receptor desensitization, internalization, and ERK1/2 phosphorylation is considered. In accordance with previous observations made for cimetidine, ranitidine and tiotidine, famotidine-induced H2R desensitization does not involve GRKs participation. Moreover, receptor internalization appeared to mediate receptor down-regulation rather than recycling, opposed to that observed for the agonist amthamine (Fernandez et al., 2008). These findings support that inverse agonist induced receptor desensitization/internalization, receptor-partners profile, and receptor cellular fate once the receptor is internalized, is strikingly different from that observed when the process is triggered by agonists. Although ligand efficacies are shared, the involved mechanisms differ, supporting that receptor partners engaged in a certain pathway are strongly dependent on the ligand, adding an additional level of ligand functional selectivity. This phenomenon was also observed for antipsychotic drugs that acting as serotonin antagonists lead to receptor desensitization and internalization (Gray and Roth, 2001).

**FIGURE 7:**
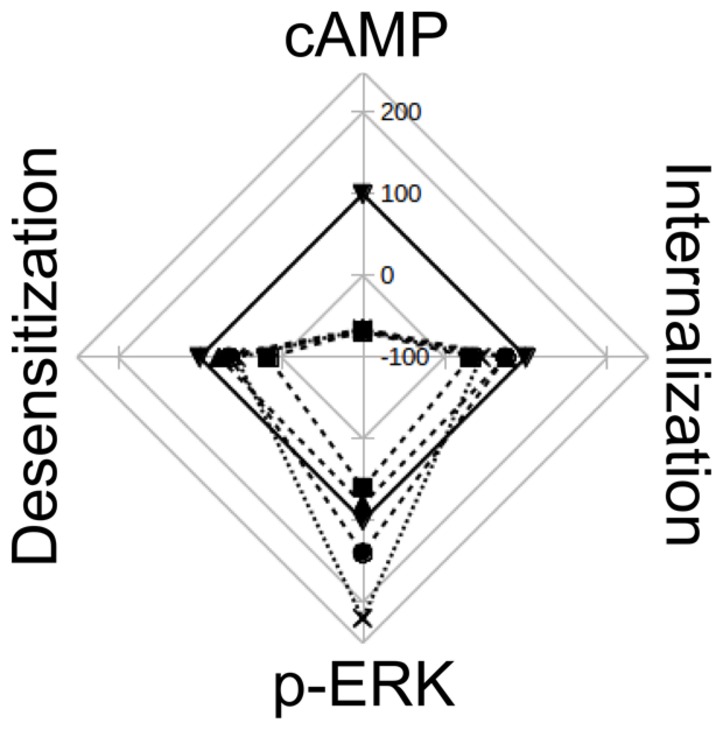
Web of efficay. The scheme represents the efficacies of amthamine (▼), cimetidine (■), ranitidine (∙), tiotidine (♢), and famotidine (x), on cAMP accumulation, pERK levels, receptor desensitization and internalization. Values are percentages relatives to amthamine efficacy for each receptor behavior. The web represents Emax values on a scale from -100 (center) to 250 (exterior line), with intervals of 50.

Webs of potencies and efficacies are a very graphical and informative way of depicting similarities and differences in pharmacological profiles between various ligands (Evans et al., 2010; Reher et al., 2012). **Figure 7** was made collecting data from this work and from bibliography, and represents the relative efficacy in HEK293T cells of diverse H2R ligands respect to amthamine. It can be clearly seen that while amthamine can be referenced as a “balanced” ligand, H2 inverse agonists have an evident bias toward ERK pathway.

Several decades ago there has been a revolution for the treatment of acid-related disorders. With the description of the involvement of histamine on gastric acid secretion and the availability of safe antagonists, H2R blockers became of the top marketed drugs around the globe. Various preparations of famotidine are available over the counter in various countries and for decades its use alleviated the symptoms of many people. Till now, accumulated clinical evidence confirms that famotidine is well tolerated and does not have any of the antiandrogenic effects reported with cimetidine. Furthermore, it does not alter the hepatic metabolism of drugs, making of famotidine an effective, well-tolerated and more potent alternative to cimetidine and ranitidine.

In the 1980s, the discovery of proton pumps for controlling gastric acid secretion made of the proton pumps inhibitors the drugs to emerge as the treatment of choice for acid-related diseases. Current guidelines indicate that PPIs should be the first drug treatment, because they are more effective than H2R blockers. Because of this, H2R blockers almost fell into disuse. However, there are present recommendations for a profylactic use of H2R antagonists in patients taking NSAIDs to prevent GI ulcers. As already mentioned in the introduction, a fixed-dose combination of ibuprofen, and famotidine (DUEXIS^®^) is available for the symptomatic treatment of arthritic symptoms and also to reduce the risk of GI ulcers in patients requiring ibuprofen therapy. The use of the combination of these two drugs was shown to reduce the risk of ulcers by 50% compared with ibuprofen alone (Laine et al., 2012).

Since the recognition of ligand bias, pharmacologists have speculated that using biased ligands could achieve novel pharmacological effects distinct from classical interventions (Kenakin, 2007). In general, the potential beneficial effects have been emphasized. Biased ligands could surpass on-target adverse events by avoiding undesirable signaling pathways, or increase their efficacy by avoiding or stimulating specific positive or negative feedback loops in signaling pathways. Numerous examples of each have been proposed. However, here we found a case where ligand bias may drive and be the cause of unwanted side-effects. The observations made in AGS cells, that endogenously express H2R, suggest that the pluridimensionality of signaling efficacies may be extended to naïve cells making our findings pharmacologically relevant. Recent data provide evidence of the existence of ligand-specific H2R conformations that explain the differences among these ligands’ affinities, potencies and efficacies observed in neutrophils and eosinophils (Reher et al., 2012). In our work, we show that famotidine, an H2R inverse agonist whose clinical use lies on the blocking of histamine, actually mimics the effects of the natural agonist concerning G-protein independent signaling pathways. In this regard, ERK1/2 activation and the induction of HDC gene expression can be envisaged as an undesired effect, since the treatment with the inverse agonist may induce the same effects that the agonist that is intended to block, and induces the expression of the enzyme that synthesizes the natural agonist, potentially increasing the circulating levels of the ligand that should antagonize. HDC promoter activity is upregulated by gastrin, *Helicobacter pylori* and PACAP, all causing acid-related disorders. On the other hand, targeted gene disruption of HDC and H2R, demonstrate the key role of gastric acid secretion mediated by hormones such as gastrin or PACAP (Chen et al., 2006).

Prolonged H2R blockade was found to increase parietal cell sensitivity to H2 agonists and tolerance to the treatment. This was explained in terms of receptor upregulation due to structural stabilization of the receptor by the inverse agonists (Smit et al., 1996). In this context, the puzzling effect of famotidine over HDC expression could be a supplementary explanation regarding the undesired effects observed after withdrawal of H2R blockers, and may also explain why these ligands produce rebound acid hypersecretion after withdrawal (Smith et al., 1999). It can be expected that after famotidine treatment, histamine secretion would be augmented causing a new outbreak of clinical manifestations. The resuming of the treatment should alleviate the symptoms, however, patients are advised not to immediately resume treatment, as rebound symptoms are spontaneously reversible and are likely to improve within a few days.

Cumulative knowledge demonstrates that there is a need for the medical community to understand and continue to study biased agonism due to its potential clinical relevance. Considering current clinical use of biased agonists/antagonists, it is important for physicians and pharmacists to understand which drugs are biased agonists and in which cases they should be used and avoided.
